# An association between autosomal-dominant polycystic kidney disease and the risk of acute myocardial infarction in Asian population — results of a nationwide study

**DOI:** 10.18632/oncotarget.14269

**Published:** 2016-12-27

**Authors:** Pei-Hsun Sung, Hsin-Ju Chiang, Yao-Hsu Yang, Chi-Jen Chen, John Y. Chiang, Hon-Kan Yip

**Affiliations:** ^1^ Division of Cardiology, Department of Internal Medicine, Kaohsiung Chang Gung Memorial Hospital and Chang Gung University College of Medicine, Kaohsiung, Taiwan; ^2^ Department of Obstetrics and Gynecology, Kaohsiung Chang Gung Memorial Hospital and Chang Gung University College of Medicine, Kaohsiung, Taiwan; ^3^ Department for Traditional Chinese Medicine, Chang Gung Memorial Hospital, Chiayi, Taiwan; ^4^ Institute of Occupational Medicine and Industrial Hygiene, National Taiwan University College of Public Health, Taipei, Taiwan; ^5^ Center of Excellence for Chang Gung Research Datalink, Chang Gung Memorial Hospital, Chiayi, Taiwan; ^6^ Department of Computer Science and Engineering, National Sun Yat-Sen University, Kaohsiung, Taiwan; ^7^ Department of Healthcare Administration and Medical Informatics, Kaohsiung Medical University, Kaohsiung, Taiwan; ^8^ Institute for Translational Research in Biomedicine, Center for Shockwave Medicine and Tissue Engineering, Kaohsiung Chang Gung Memorial Hospital and Chang Gung University College of Medicine, Kaohsiung, Taiwan; ^9^ Department of Medical Research, China Medical University Hospital, China Medical University, Taichung, Taiwan; ^10^ Department of Nursing, Asia University, Taichung, Taiwan

**Keywords:** autosomal-dominant polycystic kidney disease, acute myocardial infarction, Asian, Taiwanese, population-based cohort study

## Abstract

Cardiovascular complications are the leading causes of death in patients with autosomal-dominant polycystic kidney disease (ADPKD) in the Western countries. However, theprevalence and risk of acute myocardial infarction (AMI) in patients with ADPKD remain unknown, especially in Asian population. We utilized the data from Taiwan National Health Insurance Research Database (NHIRD) to perform a population-based cohort study (1997-2008). A total of 2062 patients with ADPKD were selected from one million of general population after excluding those patients with age less than 18 years old, receiving renal replacement therapy, and concomitant diagnoses of AMI. Additionally, we set up those patients without ADPKD as comparison group by matching study cohort with age, gender, income and urbanization with 1:10 ratio (n=20620). The results showed that although the prevalence of AMI in ADPKD patients in Taiwan was lower than those in the United States (2.91% *v.s*. 6%, *p*=0.0567), the Taiwanese ADPKD group had significantly higher prevalence of AMI as compared with the non-ADPKD group (2.91% *v.s*. 0.97%, *p*<0.0001). In addition, Kaplan-Meier analysis demonstrated that cumulative incidence of AMI was significantly higher in ADPKD than in the non-ADPKD group (all *p*<0.001). After adjusting for age, gender and comorbidities by multivariate and sensitivity analysis, ADPKD patients had 2.43-fold greater risk for developing AMI as compared with non-ADPKD patients (95% CI 1.8 to 3.29, *p*<0.0001). In conclusion, Taiwanese patients with ADPKD have lower prevalence of AMI as compared to Americans, whereas ADPKD *per se* remains independently predictive of AMI in Asian population.

## INTRODUCTION

Autosomal-dominant polycystic kidney disease (ADPKD) is the most common hereditary kidney disease [1]. The heterogeneous mutation of PKD 1 and PKD2 genes contributes to the development of ADPKD [2]. Prevalence of ADPKD in the United States has been reported to be around 1/1000 to 1/400 [3]. Even though the appropriate treatment was applied, end-stage of renal disease (ESRD) has been revealed to be eventually developed in 8%-10% of patients with ADPKD in the United States and Europe [4]. Additionally, as compared with the general population, patients with ADPKD have 1.6 to 3.2-folds of relative morality rate [5].

Because of higher frequency of early-onset hypertension, left ventricular hypertrophy and valvular abnormalities, cardiovascular (CV) complications are the most common cause of death in the APDKD population [6–8]. In fact, 81% of ADPKD patients have been reported to have coronary artery disease [8]. Furthermore, recent registered data has demonstrated that the prevalence rate of acute myocardial infarction (AMI) in American ADPKD patients was up to 6% [9]. Therefore, it deserved to be further understood how to prevent, diagnose and treat such a medical urgency in the ADPKD population.

Intriguingly, while an association between ADPKD and CV diseases including AMI had been well recognized in Western countries [6–9], there was no data to address the incidence and risk of AMI in ADPKD in Asia. Additionally, little is known whether prevalence of AMI in Asian ADPKD patients is actually as high as that in the Western population. This is mainly due to lack of long-term follow-up and surveillance of the CV complications in patients with ADPKD.

By using a 12-year Taiwan National Health Insurance Research Database (NHIRD) [10, 11], we intended to study the real-world prevalence, incidence and associated risk of AMI in Asian ADPKD patients.

## RESULTS

### Demographic characteristics, comorbidities, and prevalence of AMI in Taiwanese patients with and without ADPKD (Table 1)

Table 1 shows comparison of baseline characteristics and comorbidities between study and comparison cohorts. A total of 2,062 patients with ADPKD and 20,620 matched patients without ADPKD were eligible during 12-year dataset period. In both groups of ADPKD and non-ADPKD, 51.5% patients were female and median age was 47 years old (interquartile range 38-56). Majority of the patients were middle-aged (40-65 years old, 61.2%) and had urbanization level 1-2 (80.21%). Additionally, most of their economic status was relatively poor (i.e., monthly insurance taxable income ≤25,000 New Taiwan dollars) (72.54%). However, the frequency of comorbidities was significantly higher in the ADPKD group than that in the non-ADPKD group (all *p*-values <0.002), except for diabetes mellitus (14.99% *v.s*. 15.15%, *p*=0.8467).

**Table 1:** Demographic characteristics, comorbidities, and prevalence of AMI in Taiwanese patients with ADPKD and without ADPKD

	ADPKD (N = 2062)		Non-ADPKD* (N = 20620)		P-value^a^
	No.	%	No.	%	
Gender					1.00
Female	1062	51.5	10620	51.5	
Male	1000	48.5	10000	48.5	
Age					1.00
18-39	574	27.84	5740	27.84	
40-65	1262	61.2	12620	61.2	
>65	226	10.96	2260	10.96	
Median age (IQR)	47 (38-56)		47 (38-56)		
Urbanization					1.00
1 (highest)	742	35.98	7420	35.98	
2	912	44.23	9120	44.23	
3	291	14.11	2910	14.11	
4 (lowest)	117	5.67	1170	5.67	
Monthly income (NTD)					1.00
0	343	16.63	3430	16.63	
1-15,840	296	14.35	2960	14.35	
15,841-25,000	857	41.56	8570	41.56	
>25,000	566	27.45	5660	27.45	
Comorbidities					
Hypertension	1672	81.09	6523	31.63	<.0001
Diabetes mellitus	309	14.99	3123	15.15	0.8467
Dyslipidemia	714	34.63	4499	21.82	<.0001
Atrial fibrillation	49	2.38	301	1.46	0.0013
Ischemic heart disease	537	26.04	3257	15.8	<.0001
Heart failure	217	10.52	833	4.04	<.0001
Peripheral vascular disease	136	6.6	668	3.24	<.0001
Chronic kidney disease	1011	49.03	452	2.19	<.0001
Malignancy of kidney or bladder	65	3.15	93	0.45	<.0001
Acute myocardial infarction					<.0001
No	2002	97.09	20419	99.03	
Yes	60	2.91	201	0.97	

* Control group (non-ADPKD group) was matched by age, sex, monthly income and urbanization level.

^a^Chi-square test for categorical variables

AMI = acute myocardial infarction, ADPKD = autosomal-dominant polycystic kidney disease,

IQR = Interquartile range, NTD = New Taiwan dollars.

At the end of follow-up period, a total of 60 and 201 cases of AMI developed in 2,062 ADPKD and 20,620 non-ADPKD patients, respectively. Accordingly, the ADPKD group had significantly higher prevalence rate of AMI as compared with the non-ADPKD group in Taiwan (2.91% *v.s*. 0.97%, *p*<0.0001).

### Ethnic difference of prevalence of AMI and relevant cardiovascular comorbidities between American and Taiwanese patients with ADPKD (Table 2)

Data from Taiwan NHIRD reported the prevalence of AMI in ADPKD was 2.9%. On the other hand, the Americanregistered data from Helal et al.[9] revealed that the prevalence of AMI in American ADPKD population was 6%, suggesting that the prevalence of AMI in ADPKD was relatively lower in Asian than in Western population (*p*=0.056). Additionally, the prevalence of hypertension, dyslipidemia, arrhythmia, valvular heart disease and peripheral vascular disease in ADPKD was significantly lower in Taiwanese than in American population (all *p*-valve <0.002). Contrast to these parameters, the prevalence of heart failure in ADPKD was similar between these two different ethnical populations (i.e., around 10% in both, p=0.5527). It was noteworthy that Taiwanese ADPKD population had much higher prevalence of diabetes mellitus than American ADPKD population (15% *v.s*. 8.7%, *p*=0.0008).

**Table 2:** Comparison of prevalence of AMI and relevant cardiovascular comorbidities between American and Taiwanese patients with ADPKD

	American (N = 419)*	Taiwanese (N = 2062)	P-value
Age	53.2 ± 13.7	47.6 ± 13.6	
Gender			<.0001
Female	64.6% (265/410)	51.5%	
Male	35.4% (145/410)	48.5%	
Acute myocardial infarction	6% (24/399)	2.9%	0.0567
Comorbidities			
Hypertension	86.6% (356/411)	81.1%	0.0081
Diabetes mellitus	8.7% (36/412)	15.0%	0.0008
Dyslipidemia	45.7% (188/411)	34.6%	<.0001
Arrhythmia	25.9% (103/398)	19.3%	0.0028
Heart failure	9.5% (38/400)	10.5%	0.5527
Valvular heart disease	14.4% (57/397)	8.5%	0.0003
Peripheral vascular disease	16.5% (66/400)	6.6%	<.0001

AMI = acute myocardial infarction, ADPKD = autosomal dominant polycystic kidney disease

*Data was adopted from the publication by Helal et al.[9]

### Comparison of incidence and associated risk of AMI between Taiwanese patients with ADPKD and without ADPKD (Table 3)

The incidence rate of AMI in patients with and without ADPKD was 459.5 and 145.1 per 100,000, respectively. Therefore, incidence rate ratio (IRR) of AMI in ADPKD to non-ADPKD was 3.17 (95% CI 2.37 to 4.22, *p*<0.0001). After adjusting for age, gender and comorbidities with multivariate Cox regression analysis, patients with ADPKD had 2.43-fold risk for new occurrence of AMI as compared with those without ADPKD (95% CI 1.8 to 3.29, *p*<0.0001). Besides, stratified analysis which was utilized to clarify the impact of each comorbidity on AMI showed that adjusted hazard ratio of AMI in ADPKD to non-ADPKD group was significantly higher in the subgroups of both gender, age older than or equal to 40 years old, and those with traditional atherosclerotic risk factors, i.e., hypertension, diabetes and dyslipidemia (all *p*-values <0.03). These findings suggested the risk of AMI in ADPKD was actually greater than in non-ADPKD, especially in those patients aged ≥40 years old with any one of traditional risk factors of atherosclerosis. Moreover, as to those subgroups void of atrial fibrillation, heart failure, peripheral vascular disease, chronic kidney disease and urogenital malignancy, ADPKD was still identified as an independent predictor for future development of AMI (all *p*-values <0.0001).

**Table 3:** Comparison of incidence and hazard ratio of AMI between patients with and without ADPKD, stratified by gender, age and comorbidities

Variables	ADPKD						IRR (95% CI)	Adjusted HR (95% CI)
	No (N = 20620)			Yes (N = 2062)				
	Event	PY	Rate	Event	PY	Rate		
Acute myocardial infarction	201	138508	145.1	60	13059	459.5	3.17 (2.37 - 4.22)***	2.43 (1.8 - 3.29)***
Sex								
Female	67	72435	92.5	21	6923	303.3	3.28 (2.01 - 5.35)***	2.7 (1.62 - 4.47)***
Male	134	66073	202.8	39	6136	635.6	3.13 (2.19 - 4.48)***	2.3 (1.58 - 3.35)***
Age								
18-39	9	38312	23.5	2	3788	52.8	2.25 (0.49 - 10.4)	1.22 (0.19 - 8.03)
40-65	114	85756	132.9	45	8125	553.8	4.17 (2.95 - 5.88)***	2.74 (1.91 - 3.94)***
>65	78	14440	540.2	13	1146	1134.4	2.1 (1.17 - 3.78)*	1.95 (1.08 - 3.51)*
Comorbidities								
Hypertension								
No	41	91966	44.6	5	2129	234.9	5.27 (2.08 - 13.33)***	6.49 (2.52 - 16.7)***
Yes	160	46542	343.8	55	10930	503.2	1.46 (1.08 - 1.99)*	2.21 (1.61 - 3.03)***
Diabetes mellitus								
No	113	116325	97.1	45	11138	404	4.16 (2.94 - 5.88)***	2.64 (1.83 - 3.83)***
Yes	88	22183	396.7	15	1921	780.8	1.97 (1.14 - 3.4)*	2.05 (1.18 - 3.56)*
Dyslipidemia								
No	114	106456	107.1	33	8513	387.6	3.62 (2.46 - 5.33)***	2.49 (1.66 - 3.74)***
Yes	87	32052	271.4	27	4546	593.9	2.19 (1.42 - 3.37)***	2.29 (1.46 - 3.59)***
Atrial fibrillation								
No	181	136277	132.8	55	12713	432.6	3.26 (2.41 - 4.4)***	2.54 (1.85 - 3.49)***
Yes	20	2231	896.5	5	346	1445.1	1.61 (0.61 - 4.3)	1.57 (0.56 - 4.39)
Ischemic heart disease								
No	0	115228	0	0	9556	0	---^†^	---
Yes	201	23280	863.4	60	3503	1712.8	1.98 (1.49 - 2.65)***	2.17 (1.61 - 2.93)***
Heart failure								
No	142	132487	107.2	41	11639	352.3	3.29 (2.32 - 4.65)***	2.57 (1.78 - 3.71)***
Yes	59	6021	979.9	19	1420	1338	1.37 (0.81 - 2.29)	1.49 (0.86 - 2.56)
Peripheral vascular disease								
No	185	133663	138.4	54	12072	447.3	3.23 (2.39 - 4.38)***	2.42 (1.76 - 3.33)***
Yes	16	4845	330.2	6	987	607.9	1.84 (0.72 - 4.7)	2.65 (0.96 - 7.25)
Chronic kidney disease								
No	178	135392	131.5	21	6159	341	2.59 (1.65 - 4.08)***	2.82 (1.76 - 4.53)***
Yes	23	3116	738.1	39	6900	565.2	0.77 (0.46 - 1.28)	1.14 (0.65 - 2)
Malignancy of kidney or bladder								
No	196	137883	142.1	59	12620	467.5	3.29 (2.46 - 4.4)***	2.55 (1.88 - 3.47)***
Yes	5	625	800	1	439	227.8	0.28 (0.03 - 2.44)	---

* *p*<0.05, **p<0.01, ***p<0.001

†Insufficient case number for statistical analysis

Rate denotes incidence rate (per 100,000).

Adjusted HR (hazard ratio) indicates multivariate analysis with adjusting for age, gender, and comorbidities.

AMI = acute myocardial infarction, ADPKD = autosomal dominant polycystic kidney disease,

PY = person-year, IRR = incidence rate ratio, CI = confidence interval.

### Cox regression analysis for identification of the independent risks of AMI (Table 4)

By using the univariate Cox regression analysis, we found that the risk of AMI significantly increased with age. Additionally, male gender, ADPKD, risk factors of atherosclerosis (i.e., hypertension, diabetes mellitus, and dyslipidemia) and each of comorbidities were also identified as the independent risk factors for AMI. After adjusting for age, gender and comorbidities with multivariate model, ADPKD *per se* remained a powerful predictor for future development of AMI (adjusted HR 2.09, 95% CI 1.41 to 3.1, *p*=0.0003). Besides, we also found that male gender, older age, hypertension, diabetes mellitus, and heart failure were strongly and independently predictive of AMI (all *p*-valves <0.0007).

**Table 4:** Cox proportional hazard regression model of predictors for new occurrence of AMI

	Univariate			Multivariate		
	HR	95% CI	P-value	HR	95% CI	P-value
Gender						
Female	1.00			1.00		
Male	2.17	1.68 - 2.81	<.0001	2.4	1.83 - 3.15	<.0001
Age						
18-39	1.00			1.00		
40-65	6.43	3.49 - 11.84	<.0001	4.11	2.19 - 7.72	<.0001
>65	22.24	11.9 - 41.58	<.0001	7.14	3.67 - 13.9	<.0001
Urbanization						
1 (highest)	1.00			1.00		
2	0.88	0.67 - 1.17	0.3754	0.81	0.61 - 1.07	0.1319
3	1.13	0.78 - 1.62	0.5223	0.9	0.62 - 1.32	0.5984
4 (lowest)	1.85	1.18 - 2.9	0.007	1.29	0.81 - 2.07	0.2837
Monthly income (NTD)						
0	1.00			1.00		
1-15,840	1.2	0.81 - 1.79	0.3617	1.24	0.83 - 1.86	0.2921
15,841-25,000	0.88	0.62 - 1.24	0.4566	0.91	0.63 - 1.32	0.6277
>25,000	0.59	0.4 - 0.88	0.0095	0.69	0.45 - 1.08	0.1018
ADPKD						
No	1.00			1.00		
Yes	3.21	2.4 - 4.28	<.0001	2.09	1.41 - 3.1	0.0003
Comorbidities						
Hypertension	7.53	5.47 - 10.35	<.0001	3.08	2.14 - 4.41	<.0001
Diabetes mellitus	3.4	2.65 - 4.36	<.0001	1.62	1.23 - 2.12	0.0006
Dyslipidemia	2.4	1.88 - 3.07	<.0001	1.25	0.96 - 1.63	0.1039
Atrial fibrillation	6.03	3.99 - 9.11	<.0001	1.4	0.89 - 2.19	0.1436
Heart failure	8.1	6.21 - 10.56	<.0001	3.09	2.27 - 4.2	<.0001
Peripheral vascular disease	2.26	1.46 - 3.5	0.0002	0.84	0.53 - 1.31	0.4366
Chronic kidney disease	4.39	3.3 - 5.84	<.0001	1.23	0.83 - 1.82	0.3007
Malignancy of kidney or bladder	3.35	1.49 - 7.53	0.0034	1.27	0.56 - 2.89	0.573

AMI = acute myocardial infarction, HR = hazard ratio, CI = confidence interval, NTD = New Taiwan dollars,

ADPKD = autosomal dominant polycystic kidney disease

### Occurrence of AMI in relation to length of time since diagnosis of ADPKD (Figure 1)

The occurrence of AMI increased steadily with time in both ADPKD and non-ADPKD groups. However, the Kaplan-Meier analysis demonstrated that the cumulative incidence of AMI was significantly higher in the ADPKD group than in the non-ADPKD group (by Log-Rank test, *p*<0.001).

**Figure 1:**
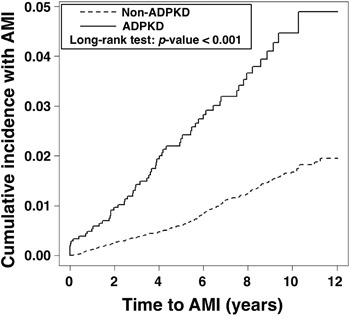
Cumulative incidence of acute myocardial infarction in the ADPKD group versus the non-ADPKD group ADPKD = autosomal-dominant polycystic kidney disease

## DISCUSSION

Previous studies emphasized on the high prevalence of CV morbidities and mortalities in ADPKD [4, 6, 9]. Additionally, the prevalence of ADPKD has been established to be 1 to 2.5 in 1,000 Caucasian general populations [3]. However, these data were only analyzed from the database of Western countries [4, 6, 9]. Accordingly, the real-world association between AMI and ADPKD in the Asian population remains unclear. The present study utilizing Taiwan NHIRD was the first one to find that the prevalence of ADPKD in Asian population (i.e., approximately 2.1 in 1,000 Taiwanese individuals) is very similar to that in Western countries. Additionally, the prevalence of AMI in the Taiwan ADPKD population was approximate 3% (Tables 1 and 2). Furthermore, the ADPKD *per se* possessed two-fold risk for development of AMI in the future. Elderly male ADPKD patients with atherosclerotic risk factors might have higher risk for event of AMI. Moreover, male gender, older age, hypertension, diabetes and heart failure were also identified as the independent risk factors for future development of AMI in ADPKD. Most importantly, to the best of our knowledge, this is the first and biggest nationwide population-based cohort study to establish a strong association between AMI and ADPKD in Asian population.

Fick et al. previously investigated the causes of death in ADPKD by using the data from autopsies [6] and the results demonstrated that up to 89% of the autopsied patients had history of cardiac hypertrophy and 81% of them had history of coronary artery disease. Besides, moderate to severe arteriosclerosis was also commonly found in aorta, suggesting that left main ostial stenosis or aortic disease might occur in patients with ADPKD. Furthermore, Helal et al. has recently reported that the prevalence of CV events in ADPKD was common, ranging from 25.9% for arrhythmia to 5% for brain aneurysm [9]. These data [6, 9] and previous literature reviews [4, 7, 12] implied that cardiovascular complications have emerged as a major cause of death in patients with ADPKD, especially in those patients with left ventricular hypertrophy (i.e., the most powerful predictor for CV morbidity and mortality), intracranial and extracranial aneurysms, as well as coronary artery diseases. In addition, data from our study also showed that the frequency of hypertension, diabetes, and dyslipidemia was notably higher in ADPKD than in non-ADPKD group. Accordingly, the aforementioned and our studies [4, 6, 7, 9, 12] highlighted that to early recognize ADPKD and aggressively control the blood pressure could be the better way to prevent most of these CV abnormalities and slow-down renal function deterioration to end-stage renal disease. Unfortunately, even though abundant evidence had shown that Renin-Angiotensin-Aldosterone System (RAAS) blockade and statin are effective to treat chamber hypertrophy, vascular remodeling, and endothelial dysfunction in ADPKD [13–15], death from AMI is always unpredictable and catastrophic. Therefore, to further understand the incidence and risk of AMI in ADPKD is a very important clinical issue.

Apart from traditional risk for coronary atherosclerosis, e.g., advancing age, male gender, hypertension, diabetes, dyslipidemia, and so on, ADPKD *per se* was also identified as a risk factor for AMI in the present study. The potential pathophysiology was supposed to be related to intracoronary aneurysm, spontaneous coronary dissection, and early-onset endothelial dysfunction [4, 16–18]. The prevalence of AMI in ADPKD patients has been well recognized to be 6% in the United States by previous study [9]. On the other hand, the prevalence of AMI in middle-aged general population was estimated of 2.03% [19], suggesting that AMI is a common and severe CV complication of ADPKD in the Western countries (i.e., 6% *v.s*. 2.03%). Similarly, the essential findings from our study demonstrated that as compared with general population (i.e., non-ADPKD) in Taiwan, patients with ADPKD had significantly higher prevalence of AMI (i.e., 2.91% *v.s*. 0.97%, p <0.0001). Besides, our study found that ADPKD *per se* carried two-fold risk for occurrence of AMI. Therefore, ADPKD indeed should be recognized as an independent risk factor for future development of AMI all over the world. This finding strengthens again the importance of early and vigorous controlling underlying diseases, especially for hypertension, in any ADPKD patient. Most importantly, heart attack had to always be taken into consideration for those patients with ADPKD complaining any chest discomfort.

The prevalence of AMI in ADPKD in Taiwan was lower than in America (i.e., 2.9% *v.s*. 6%, *p*=0.056) [9]. However, we remain uncertain for why the different prevalence of AMI in ADPKD is present between Asian and Western population. Perhaps, it is due to the ethnical disparity, environment exposure, or significantly lower prevalence of dyslipidemia in Taiwanese as compared to that in Western ADPKD population (Table 2). Interestingly, recent data from literature reviews had showed that Chinese have less prevalence of ischemic heart disease and AMI as compared to Europeans and Americans [20–23]. Therefore, the results of these epidemiological analyses supported our present findings.

### Limitations

Our study has limitations. First, detailed personal history and lifestyle information such as smoking, body mass index, and functional capacity are not provided by Taiwan NHIRD. Second, all the data in the current study have been registered with ICD-9-CM codes, therefore further classification of disease status and determination of disease lesion is impracticable. Third, the laboratory data are not available in NHIRD. Fourth, this study did not investigate the incidence of cerebral vascular accidence in ADPKD patients. Finally, the impact of medications, e.g., RAAS blockades and statins, on ADPKD patients was not analyzed in the present study.

## MATERIALS AND METHODS

### Data source

The National Health Insurance (NHI) program in Taiwan provides health care to 99% of the 23.74 million population and links 97% of the hospitals and clinics in Taiwan (http://nhird.nhri.org.tw/en/) [11]. The researchers are able to register and claim data of 1,000,000 individuals who have been systematically selected from all insured enrollees of the National Health Research Institute (NHRI) data bank. The NHI dataset included robust information regarding medical facilities, details of inpatient and outpatient orders, dental services, prescription of drugs, patient care provided by physicians, and the scrambled registration files (e.g., payment, regions, catastrophic illness, and so on.) other than laboratory data. Diagnoses are entered in based on the International Classification of Diseases, 9^th^ Revision, Clinical Modification (ICD-9-CM).

### Study population

**Figure 2:**
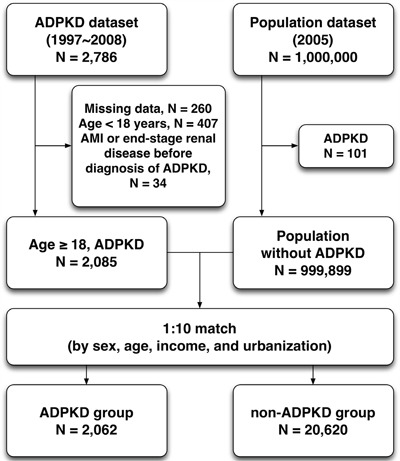
Flowchart of the patient enrollment for the ADPKD group and the matched non-ADPKD group ADPKD = autosomal-dominant polycystic kidney disease, AMI = acute myocardial infarction

This was a nationwide retrospective population-based cohort study. We selected patients with ADPKD (ICD-9-CM codes: 753.1) from 1,000,000 individuals in Taiwan NHIRD from January 1997 to December 2008. After excluding patients with follow-up duration of less than one year, missing data on baseline characteristics, age of less than 18 years, and initially concomitant diagnoses of AMI (ICD-9-CM codes 410-411) and end-stage renal disease (V451 and 549.1), a total of 2,062 ADPKD patients were identified. The comparison cohort was selected randomly by age-, gender-, income-, and urbanization-matched individuals without history of ADPKD. The ratio of non-ADPKD to ADPKD was 10:1, and therefore 20,620 non-ADPKD patients were allocated into control group (Figure 2). Urbanization of the cities/counties was categorized into four levels (from level 1 to 4 indicating the most to the least urbanized, respectively). The insurance taxable income level per month (expressed by New Taiwan dollars, NTD) was also stratified into four classifications according to monthly salary of individual insured enrollee.

### Outcomes

The diagnoses of ADPKD and AMI were confirmed by consecutive and at least three records of outpatient visits within one year or one diagnosis on admission during study period. We also verified the accuracy of diagnosis of ADPKD by checking the registration of catastrophic illness. The date of the initial diagnosis was defined as the index date. We estimated pre-existing comorbidities for each participant with hypertension (ICD-9-CM codes 401-405), diabetes (250), dyslipidemia (272), atrial fibrillation (427.31), ischemic heart disease (410-414), heart failure (428), peripheral vascular disease (440, 443.9, 444.0, 444.2, 444.8, 444.9, 447.8, 447.9, 445.0, 445.02), chronic kidney disease (585), and malignancy of kidney and bladder (188-189). The main purpose of this study was to evaluate the incidence of AMI (410-411) more than one year after the diagnosis of ADPKD. In addition, we compared Taiwanese data with American registered data to elucidate the ethnic difference of ADPKD and its comorbidities. Furthermore, the association between ADPKD and AMI was also analyzed for clarification of real-world risk of AMI in Taiwanese patients with ADPKD.

### Statistical analysis

We compared the distribution of demographic factors and the rate of comorbidities between the study cohort (i.e., ADPKD) and matched control cohort (i.e., non-ADPKD) with the independent *t* test or Chi-square test. The incidence rate and 95% confidence intervals (95% CI) of AMI were calculated for the entire follow-up period. Besides, Chi-square test was utilized to compare prevalence of AMI and relevant cardiovascular comorbidities between American and Taiwanese patients with ADPKD. We also utilized the Kaplan-Meier method to estimate cumulative incidences and performed the Log-Rank test to examine differences between disease group and non-disease group in the cohort study. Furthermore, Cox proportional hazard regression models were used to compute the hazard ratios (HRs) and accompanying 95% CIs after adjusting for age, gender, urbanization, income and comorbidities. In order to examine potential effect modifiers, we conducted analyses categorized by groups with and without ADPKD. We also examined the outcome (i.e., occurrence of AMI) stratified by groups according to gender, age, and each of comorbidities. The sensitivity analyses were applied to evaluate the difference and consistency between ADPKD and the risk of AMI. Two-tailed *p*-value <0.05 was considered statistically significant. All the analyses were conducted using SAS statistical software (Version 9.4; SAS Institute, Cary, NC, USA).

## CONCLUSION

The results of the present study displayed that the Taiwanese patients with ADPKD had substantially higher incidence of AMI than those without ADPKD. In addition, ADPKD *per se* carried the two-fold risk for development of AMI in the future.

## Abbreviations

ADPKD: autosomal-dominant polycystic kidney disease
AMI: acute myocardial infarction
CV: cardiovascular
NHIRD: National Health Insurance Research Database
NHRI: National Health Research Institute
HRs: hazard ratios.
